# A novel combination therapy with Uridine and Praziquantel effectively alleviates schistosomiasis-induced hepatic fibrosis through promoting adipogenic differentiation

**DOI:** 10.1371/journal.ppat.1013403

**Published:** 2025-08-06

**Authors:** Xiangyu Zhou, Qingkai Xue, Chengwei Wu, Xiaojing Li, Yuyan Wang, Yang Dai, Chunrong Xiong, Ying Zhang, Yongliang Xu, Xinjian Liu, Yuzheng Huang

**Affiliations:** 1 National Health Commission Key Laboratory of Parasitic Disease Control and Prevention, Jiangsu Provincial Key Laboratory on Parasite and Vector Control Technology, Jiangsu Institute of Parasitic Diseases, Wuxi, Jiangsu, PR China; 2 Tropical Diseases Research Center, Nanjing Medical University, Wuxi, Jiangsu, PR China; 3 Experimental Center of Clinical Research, The First Affiliated Hospital of Anhui University of Chinese Medicine, Hefei, Anhui, PR China; 4 School of Public Health, Nanjing Medical University, Nanjing, Jiangsu, PR China; 5 Department of Pathogen Biology, Key Laboratory of Antibody Techniques of National Health Commission, Nanjing Medical University, Nanjing, Jiangsu, PR China; University of Wisconsin-Madison, UNITED STATES OF AMERICA

## Abstract

Schistosomiasis-induced hepatic fibrosis, a consequence of egg-induced granulomatous lesions, remains untreated by current drugs. Therefore, the development of novel antifibrosis drugs is of paramount importance. Our previous study indicated that aberrant uridine concentrations play a pivotal role in schistosomiasis-induced hepatic fibrosis. This study aimed to explore the inhibitory role of uridine in schistosomiasis-induced liver fibrosis and the regulatory mechanism of uridine on hepatic stellate cell (HSC) activation. The results indicated that uridine could inhibit schistosomiasis-induced liver fibrosis in vivo and TGF-β-induced HSC activation in vitro. Molecular docking revealed a strong interaction between uridine and the adenosine receptor A1 (ADORA1) receptor. Subsequent in vitro investigations demonstrated that uridine modulated the cAMP/PKA/CREB pathway, influencing HSC adipogenic differentiation and exerting an antifibrotic effect. In addition, compared with praziquantel (PZQ) alone, combined uridine and PZQ therapy resulted in a reduced fibrotic area and improved hepatic parameters in vivo. Our study reveals the antifibrosis mechanism of the uridine molecule, which may be a promising drug for the treatment of schistosomiasis-induced liver fibrosis.

## Author summary

Schistosomiasis is a parasitic disease that frequently leads to chronic liver fibrosis, yet effective antifibrotic therapies remain scarce. Our previous research revealed a strong association between hepatic uridine levels and disease progression. In this study, we evaluated the therapeutic potential of exogenous uridine supplementation in a mouse model of liver fibrosis induced by Schistosoma japonicum infection. Uridine treatment significantly suppressed the activation of hepatic stellate cells (HSCs) in vitro and mitigated liver fibrosis in vivo. Mechanistically, uridine was found to bind the ADORA1 receptor and inhibit the cAMP/PKA/CREB signaling pathway, thereby modulating lipid metabolism and reducing fibrotic activity. Notably, the combination of uridine with praziquantel (PZQ), the standard antiparasitic treatment, resulted in greater improvements in liver fibrosis and function than either agent alone. Our findings not only offer new insights into the antifibrotic properties of uridine but also suggest a promising host-targeted therapeutic strategy for schistosomiasis. This research may aid in the development of more effective treatments for parasitic liver diseases and contribute to global efforts to reduce the long-term burden of schistosomiasis.

## 1. Introduction

Schistosomiasis is a prevalent parasitic disease found worldwide [1]. *Schistosoma japonicum(S. japonicum)*, one of the three main species of schistosomes responsible for human infections, typically results in pathological changes, including egg granulomas, fibrosis, and tissue damage within the liver, at chronic and advanced stages [2]. At present, the treatment of schistosomiasis relies predominantly on praziquantel(PZQ), a chemical effective at killing adult worms [3]. However, liver fibrosis still occurs in some PZQ-treated patients with advanced schistosomiasis, and specific clinical treatment strategies are lacking [4]. Our previous study revealed abnormal changes in the concentration of uridine in the livers of mice with schistosomiasis by spatial metabolome analysis, which suggested that uridine depletion during liver fibrosis may be a factor in disease exacerbation [5]. However, the effects and mechanisms of uridine on schistosomiasis-induced hepatic fibrosis remain unknown.

Uridine, a pyrimidine nucleoside derived from both plant and animal origins, is a critical component of cellular nucleic acids [6]. Previous studies have identified uridine as a conserved regenerative metabolic factor involved in regeneration across various species. It has been recognized for its capacity to activate senescent human stem cells and promote the regeneration of a variety of body tissues [7]. In addition, uridine has been shown to exert anti-inflammatory and antifibrotic effects, mitigating the hepatic fibrosis induced by carbon tetrachloride, likely by modulating the dynamism of hepatic cells [8]. Therefore, exogenous uridine supplementation may be an important strategy against schistosomiasis-related liver fibrosis.

Activated HSCs are the primary effector cells involved in liver fibrosis [9], and are responsible for the secretion of excessive amounts of extracellular matrix (ECM) components, such as α-smooth muscle actin(α-SMA) and collagen, which contribute to the progression of liver fibrosis [10]. Historically, HSCs were referred to as lipocytes owing to their substantial storage of lipid droplets rich in vitamin A [11]. During activation and subsequent transformation into a myofibroblast-like phenotype, these lipid droplets are oxidized to provide energy for cell proliferation and protein synthesis [12]. Additionally, previous studies have demonstrated that ectopic activation of adipogenic transcription, such as peroxisome proliferator activated receptor-γ (PPAR-γ) and sterol regulatory element binding protein-1c (SREBP-1c), can reverse HSC activation [13,14]. Consequently, regulating the lipogenic differentiation of HSCs, which means a transition to a quiescent state dominated by the synthesis and storage of lipid droplets [15], may serve as a strategy to inhibit HSC activation.

Adenosine receptor A1(ADORA1) is a G protein-coupled receptor(GPCR) that is widely expressed in a variety of cell types, including HSCs. Studies have indicated that upon activation, ADORA1 couples with inhibitory G proteins, leading to the inhibition of adenylate cyclase and a subsequent reduction in intracellular cyclic adenosine monophosphate (cAMP) levels [16]. cAMP functions as an intracellular second messenger that activates protein kinase A (PKA), a tetrameric protein that dissociates to release catalytic subunits with kinase activity [17]. The catalytic subunits of PKA (PKAc) can phosphorylate the serine 133 residue of cAMP-response element binding protein (CREB), and phosphorylated CREB (pCREB) can activate the transcription of downstream genes, such as the cellular Fos proto-oncogene(cFOS) and the cellular Jun proto-oncogene(cJUN), which regulate the proliferation and metabolism of cells [18,19].

PZQ is a commonly used drug for treating helminths, and it is also currently the preferred drug for treating schistosomiasis [20]. Previous studies have confirmed that the treatment with traditional Chinese medicine in combination with praziquantel is superior to monotherapy, especially for the treatment of schistosomal liver fibrosis [21]. Consequently, the combination of uridine with praziquantel may enhance the therapeutic efficacy against fibrosis. However, the effectiveness of this treatment method remains unknown.

This study elucidated the critical role of uridine in mitigating liver fibrosis induced by schistosomiasis. The findings revealed that its combination with PZQ markedly enhanced its antifibrotic effect. Additionally, the ADORA1 receptor and the cAMP pathway were identified as key mediators of the antifibrotic action of uridine, with a significant impact on the adipogenic differentiation of hepatic stellate cells. Our research suggests that uridine holds promise as a novel small-molecule therapeutic for the treatment of schistosomiasis-induced liver fibrosis.

## 2. Results

### 2.1. Uridine inhibited the proliferation and activation of LX-2 cells in vitro

**Fig 1:**
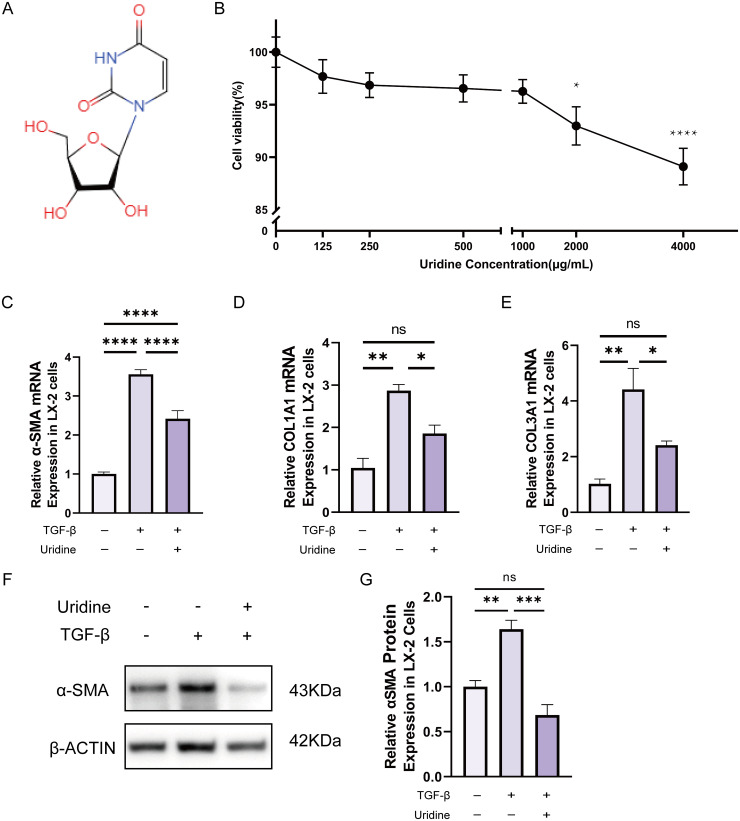
Uridine inhibited the proliferation and activation of LX-2 cells in vitro. **(A)**Chemical structure of uridine. **(B)** Cell viability of LX-2 cells treated with increasing concentrations of uridine.. (*P < 0.05, ****P < 0.0001 vs. 0 μg/mL). **(C-E)** Relative mRNA expression of α-SMA, COL1A1 and COL3A1 in LX-2 cells. **(F-G)** Representative Western blot and quantification of α-SMA expression in LX-2 cells. Data are presented as mean±SD from three independent experiments. (*P < 0.05, **P < 0.01, ***P < 0.001,****P < 0.0001).

To investigate the effect of uridine on HSCs, LX-2 cells were treated with different concentrations of uridine(structure shown in Fig 1A) in vitro. The CCK-8 results revealed that uridine markedly inhibited the proliferation of LX-2 cells in a dose-dependent manner (Fig 1B). On the basis of the CCK-8 results, 1mg/ml uridine was used to treat transforming growth factor-β (TGF-β)-induced activated LX-2 cells, and the results demonstrated that TGF-β promoted the expression of α-SMA, collagen 1A1 (COL1A1), and collagen 3A1 (COL3A1) (Fig 1C–1E), whereas uridine treatment inhibited the expression of these markers. Western blotting also verified the downregulation of α-SMA after uridine treatment (Fig 1F–1G). These results suggest that uridine can inhibit HSC activation in vitro.

### 2.2. Uridine treatment alleviated liver fibrosis in mice infected with *S.japonicum*

**Fig 2:**
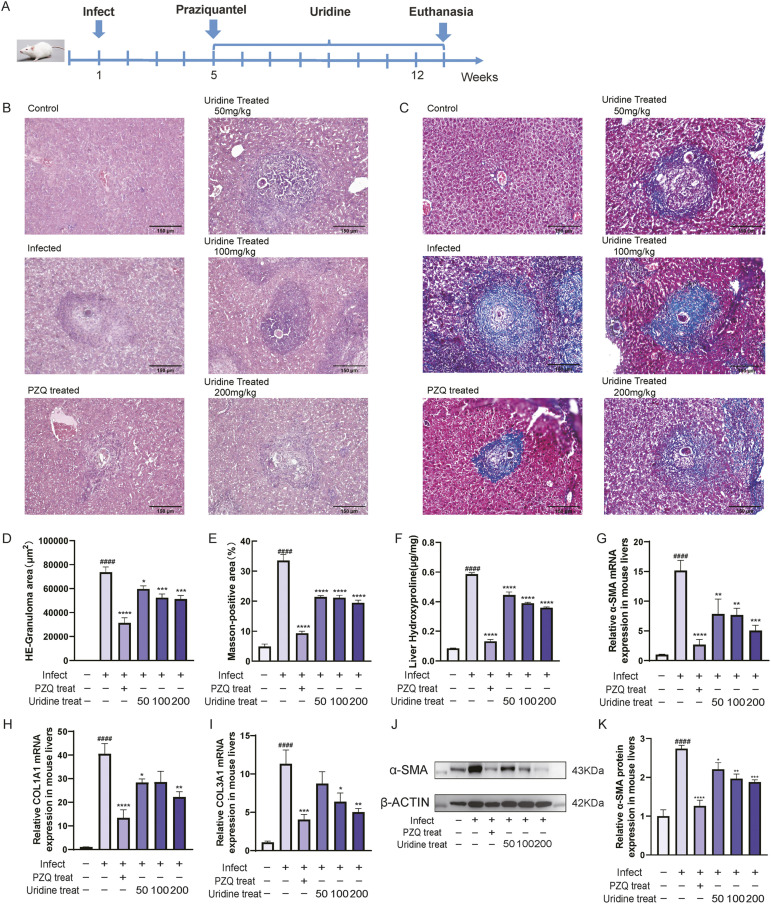
Uridine treatment alleviated liver fibrosis in mice infected with *S.japonicum.* **(A)** Schematic diagram of the experimental design of the mouse model. **(B)** Representative H&E staining images of hepatic granulomas (scale bars: 150 µm). **(C)** Fibrosis-related changes in the liver were observed by Masson staining (scale bars: 150 µm). **(D)** The areas of granulomas in the liver were analyzed with ImageJ software. **(E)** The percentage of the fibrosis area was measured by ImageJ software. **(F)** The level of hydroxyproline (HYP) in the mouse liver. (G-I) Relative mRNA expression of α-SMA, COL1A1 and COL3A1 in mouse livers. **(J-K)** Representative Western blot and quantification of α-SMA expression in mouse livers. Data represent mean±SD from five mice per group (####P < 0.0001 vs Blank control; *P < 0.05, **P < 0.01, ***P < 0.001,****P < 0.0001 vs. Infected control). The illustration in Fig 2A was drawn by hand using Adobe Illustrator 2021 software.The photos in Fig 2B and 2C were taken by the author.

To further investigate the effect of uridine on liver fibrosis in vivo, we established a mouse model of chronic liver fibrosis induced by *S. japonicum* and treated the mice orally with different doses of uridine (Fig 2A). Hematoxylin-eosin (H&E) staining of liver tissue sections revealed a reduction in inflammatory cell infiltration and hepatocyte damage in the uridine-treated group compared with the infected group (Fig 2B). In addition, Masson staining revealed that collagen fiber deposition was considerably reduced after uridine treatment in a dose-dependent manner, and the antifibrotic effect was most obvious in the 200 mg/kg group (Fig 2C). Quantitative analysis confirmed these observations, revealing a notable decrease in both the granuloma area and the hepatic fibrosis area in the uridine-treated groups (Fig 2D–2E). Additionally, the hydroxyproline (HYP) content was also reduced in the livers of uridine-treated mice, further supporting the antifibrotic effect of uridine (Fig 2F). Moreover, the expression of α-SMA, COL1A1 and COL3A1 in the livers of mice treated with a high dose of uridine was significantly lower than that in the livers of infected mice (Fig 2G–2I). Western blot analysis also revealed a progressive decrease in the expression of α-SMA(Fig 2J–2K). These results indicate that uridine treatment can markedly alleviate liver fibrosis in mice infected with *S. japonicum*.

### 2.3. Bioinformatics analysis of key targets and pathways by which uridine inhibits LX-2 cell activation in vitro

**Fig 3:**
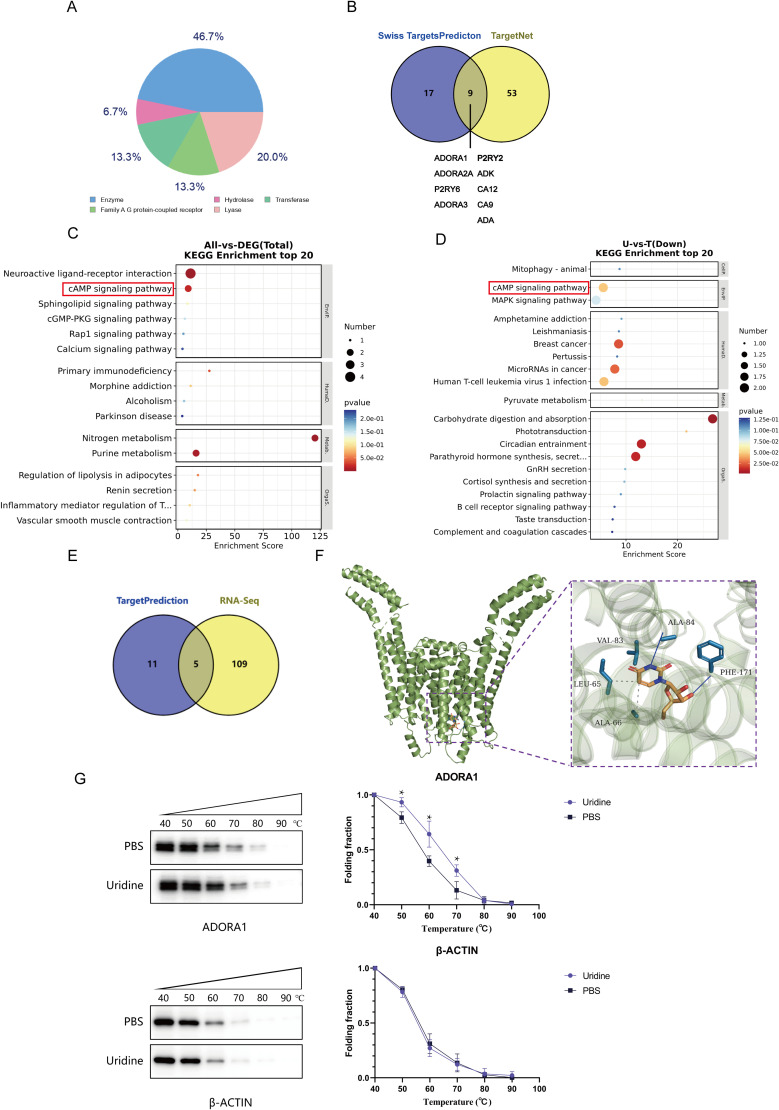
Screening of key targets and pathways by which uridine inhibits LX-2 cell activation in vitro. **(A)** The composition of the targets predicted by Swiss Target Prediction. **(B)** Venn diagram of the two sites used for predicting targets. **(C)** KEGG enrichment analysis of the predicted targets. **(D)** KEGG enrichment analysis of differentially expressed genes identified by RNA-seq (U indicates the uridine treatment group; T indicates the TGF-β treatment group). **(E)** Venn diagram of the results of the KEGG enrichment analysis of the target prediction and transcriptomics data. **(F)** Molecular docking simulation of the uridine-ADORA1 protein complex. **(G)** The thermal stability of ADORA1 in the presence of uridine. Data are presented as mean±SD from three independent experiments.. (*P < 0.05).

To further identify potential targets and pathways of uridine for the treatment of liver fibrosis, the known targets of uridine were collected from the Swiss Target Prediction and TargetNet databases. According to the statistics of the first 15 targets predicted by Swiss Target Prediction, enzymes were found to be potential targets. We focused on G protein-coupled receptors (Fig 3A). Compared with the targets predicted by TargetNet, a total of 9 common targets were confirmed by both databases (Fig 3B), and the targets were enriched by the Kyoto Encyclopedia of Genes and Genomes (KEGG) (Fig 3C). The ADORA1 receptor had the highest prediction score, we further evaluated the interaction between uridine and the ADORA1 protein through molecular docking. The docking resultsrevealed that the binding energy of the two molecules was -6.1 kcal/mol, indicating a strong interaction (Fig 3F). In addition, RNA-seq analysis was performed on TGF-β-induced activated LX-2 cells treated with uridine in vitro. Analysis of the RNA-seq data suggested that 432 genes were upregulated and that 499 genes were downregulated after uridine treatment, including the ADORA1 gene. We further analyzed the KEGG pathways of the differentially expressed genes (Fig 3D) and compared them with the KEGG enrichment results of the targets predicted by the website database. Unexpectedly, we found that the cAMP signaling pathway was coenriched by both methods (Fig 3E and S2 Table). These changes suggest that the antifibrosis function of uridine may be driven by the cAMP signaling pathway.

### 2.4. Uridine treatment downregulated the cAMP signaling pathway

**Fig 4:**
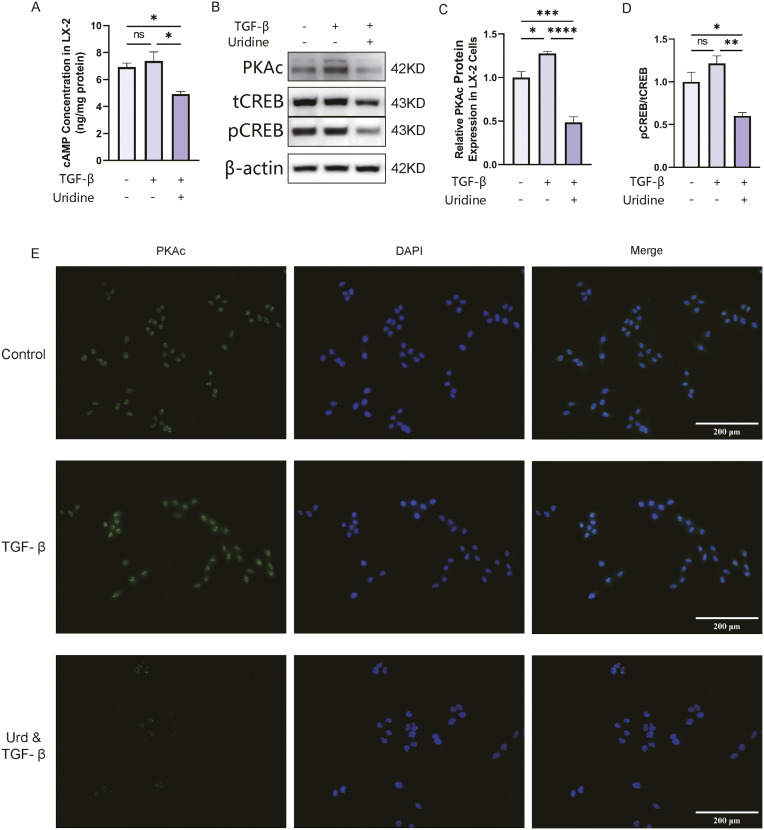
Uridine treatment inhibited the cAMP signaling pathway. **(A)** Intracellular cAMP concentration in LX-2 cells. **(B-D)** Representative Western blot (B) and quantification of Phosphokinase A protein c subunit (PKAc) (C) and CREB protein phosphorylation (D) in LX-2 cells. **(E)** Representative immunofluorescence images of PKAc (green) and nuclei (blue) in LX-2 cells. The data represent the mean±S.D from three independent experiments. (*P < 0.05, **P < 0.01, ***P < 0.001,****P < 0.0001). The photos in Fig 4E were taken by the author.

We further investigated the regulatory effect of uridine on the cAMP signaling pathway. The data demonstrated that uridine treatment notably reduced the level of the intracellular cAMP protein (Fig 4A). Moreover, TGF-β treatment upregulated PKAc protein expression, and the increase in PKAc expression increased the protein phosphorylation level of CREB and further initiated downstream gene transcription. The Western blot results revealed that the phosphorylated CREB protein in LX-2 cells tended to be increased after TGF-β treatment (Fig 4B–4D), and the immunofluorescence staining results confirmed that the protein expression of pCREB was increased in the nuclei of LX-2 cells (Fig 4E). Notably, we found that uridine treatment inhibited the expression of the cAMP signaling-related proteins PKAc and pCREB, and these results were also confirmed in in vivo experiments (S2 Fig).

### 2.5. Uridine inhibited the activation of LX-2 cells by regulating cell adipogenic differentiation through the cAMP signaling pathway

**Fig 5:**
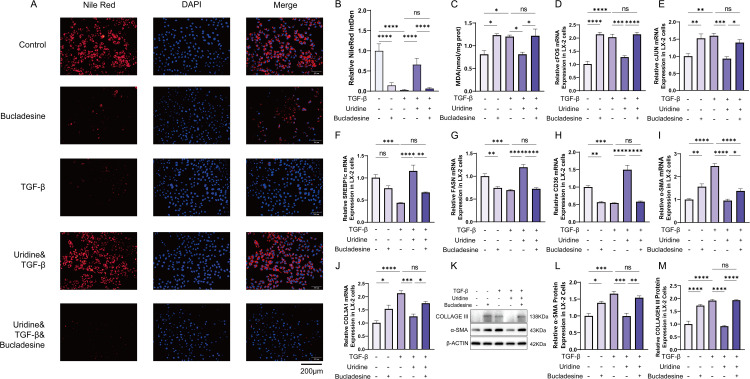
Uridine regulated cell adipogenic differentiation through the cAMP signaling pathway. **(A-B)** Representative Nile red staining image of LX-2 cells and quantification of the integrated density. **(C)** Malondialdehyde (MDA) levels in LX-2 cells. **(D-J)** Relative mRNA expression of cFOS, cJUN, SREBP1c, FASN, CD36, α-SMA and COL3A1 in LX-2 cells. **(K-M)** Representative Western blot and quantification of α-SMA and collagen III expression in LX-2 cells. The data represent the mean±S.D from three independent experiments. (*P < 0.05, **P < 0.01, ***P < 0.001,****P < 0.0001). The photos in Fig 5A were taken by the author.

Intracellular regulators of fatty acid content and metabolism play a pivotal roles in the activation of HSCs. In this study, we further investigated the effects of uridine on lipid metabolism in HSCs. Treatment with uridine enhanced lipid storage in the cytoplasm, as evidenced by increased red fluorescence intensity in Nile red staining (Fig 5A). This effect was attenuated by bucladesine, an activator of the cAMP/PKA pathway, which reduced lipid accumulation in the presence of uridine. Quantitative analysis of Nile red staining with ImageJ software confirmed the increased lipid storage in uridine-treated LX-2 cells (Fig 5B). To examine the impact of uridine on lipid oxidative decomposition, malondialdehyde (MDA) levels were measured, which were markedly reduced following uridine treatment, indicating an inhibitory effect on lipid oxidation (Fig 5C). In contrast, bucladesine reversed this inhibitory effect, leading to elevated MDA levels.

Furthermore, we assessed the transcriptional regulation of the cAMP signaling pathway targets cFOS and cJUN. Uridine treatment resulted in the downregulation of their mRNA expression (Fig 5D–5E). The addition of bucladesine restored the transcript levels of these genes, suggesting that the cAMP/PKA pathway plays a regulatory role in their expression. In line with these changes in lipid storage, uridine treatment also altered the mRNA levels of genes involved in lipid synthesis, including SREBP1c, fatty acid synthase (FASN), and CD36 (Fig 5F–5H). Additionally, the antifibrotic effects of uridine were antagonized by bucladesine, as evidenced by the upregulation of α-SMA and collagen III at both the mRNA and protein levels (Fig 5I–5M). These results collectively demonstrate that uridine inhibits LX-2 cell activation by modulating adipogenic differentiation through the cAMP signaling pathway.

### 2.6. Uridine inhibited LX-2 cell activation by regulating the ADORA1 receptor

**Fig 6:**
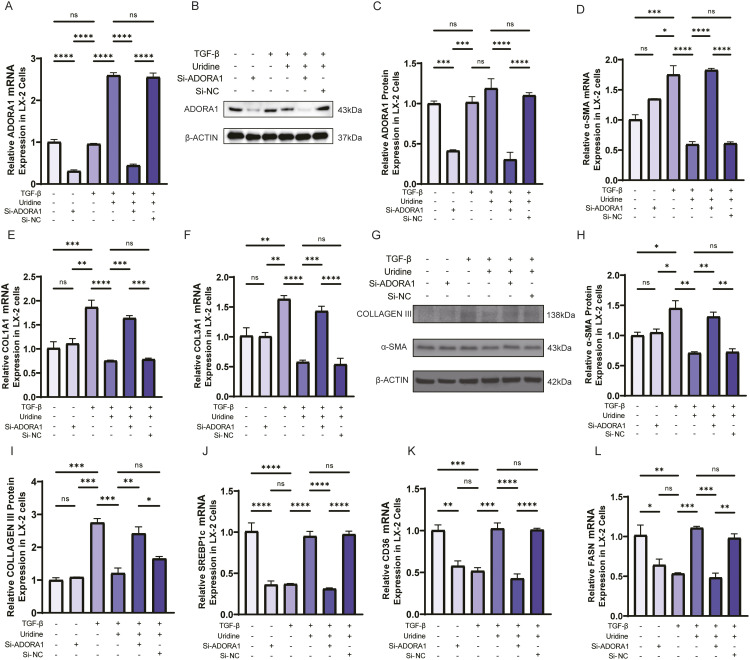
Uridine inhibited LX-2 cell activation by regulating the ADORA1 receptor. **(A)**Relative mRNA expression of ADORA1. **(B-C)** Representative Western blot and quantification of ADORA1 expression in LX-2 cells. **(D-F)** Relative mRNA expression ratio of α-SMA, COL1A1 and COL3A1. (G-I) Representative Western blot and quantification of α-SMA and collagen III expression. **(J-L)** Relative mRNA expression of SREBP1c, CD36 and FASN in LX-2 cells. The data represent the mean±S.D from three independent experiments. (*P < 0.05, **P < 0.01, ***P < 0.001,****P < 0.0001,).

Target prediction of the network database revealed that the ADORA1 receptor may be a potential target of uridine. To further evaluate the functional activity of uridine on ADORA1, we performed a cAMP-based GPCR functional assay using CHO cells. Uridine significantly reduced intracellular cAMP levels in ADORA1-knock in CHO cells in a dose-dependent manner, with an estimated EC₅₀ of 9.165 μM. No significant effect was observed in control cells lacking ADORA1 expression (S7 Fig). In addition, we found that there were no statistically significant changes in the ADORA1 gene or protein in activated LX-2 cells, but uridine treatment increased ADORA1 expression (Fig 6A–6C). To further verify the regulatory effect of uridine on ADORA1, we knocked down the expression of endogenous ADORA1 receptors with small interfering RNAs (siRNAs), and verified the knockdown effect by RT‒qPCR and Western blot(Fig 6A–6C). We subsequently analyzed the mRNA and protein levels of LX-2 cell activation markers, including α-SMA, collagen I, and collagen III. Uridine treatment substantially inhibited the activation of LX-2 cells, indicating its antifibrosis function. However, knockdown of ADORA1 counteracted these effects, leading to increased expression of fibrosis markers (Fig 6D–6I). We further examined the adipogenic phenotype of LX-2 cells, and the results revealed that uridine promoted the gene expression of SREBP1c, a major regulator of lipid synthesis, and the related proteins CD36 and FASN. Similarly, this effect of uridine was inhibited by knockout of ADORA1, suggesting that uridine regulates lipid metabolism in LX-2 cells through the ADORA1 receptor (Fig 6J–6L). These results suggest that uridine may promote the lipogenic differentiation of HSCs by modulating ADORA1 receptors, thereby inhibiting cell activation.

### 2.7. PZQ combined with uridine increased the efficacy of a single drug against schistosomiasis-induced liver fibrosis

**Fig 7:**
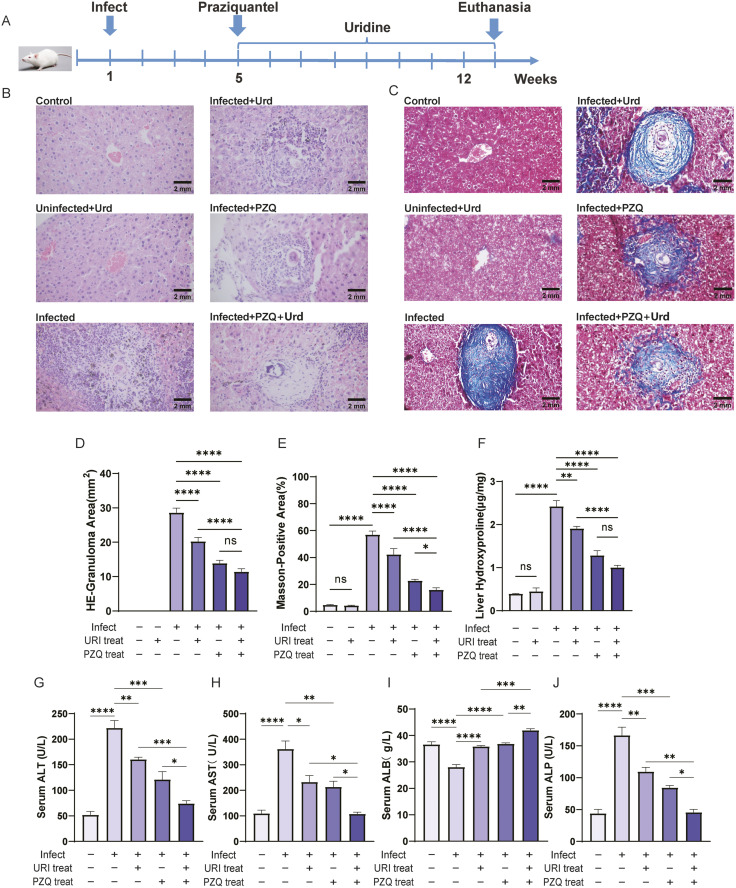
PZQ combined with uridine increased the efficacy of a single drug against schistosomiasis-induced liver fibrosis. **(A)** Schematic diagram of the experimental design of the mouse model. **(B)** Representative H&E staining images of hepatic granulomas (scale bars: 2 mm).(C) Fibrosis-related changes of the liver were observed by Masson’s trichrome staining (scale bars: 2 mm). **(D)** The area of granulomas in the liver was analyzed with ImageJ software. **(E)** The percentage of the fibrosis area was measured by ImageJ software. **(F)** HYP levels in the mouse liver. **(G-H)**Serum analyses of the hepatic parameters alanine aminotransferase (ALT), aspartate aminotransferase (AST), albumin (ALB), alkaline phosphatase (ALP). The data represent the mean±S.D from five mice per group. (*P < 0.05, **P < 0.01, ***P < 0.001, ****P < 0.0001). The illustration in Fig 7A was drawn by hand using Adobe Illustrator 2021 software.The photos in Fig 7B and 7C were taken by the author.

Given the pharmacological characteristics of PZQ, co-treatment with uridine and PZQ was applied for anti-liver fibrosis therapy. A mouse model of chronic infection with liver fibrosis induced by *S.japonicum* was established, and the oral administration of PZQ for 3 days was given as an anthelmintic therapy in the 5th week of infection, followed by the administration of oral uridine for anti-liver fibrosis treatment. H&E staining for histological examination revealed that both the uridine and PZQ treatments alone reduced the granuloma area around individual eggs in the liver tissue. However, co-treatment did not result in a statistically significant improvement compared to PZQ monotherapy in this regard (Fig 7B and 7D). On the other hand, Masson’s trichrome staining revealed that compared with either treatment alone, the coadministration of uridine with PZQ resulted in a significant decrease in the area of hepatic fibrosis (Fig 7C and 7E). Furthermore, hydroxyproline was quantified in liver tissue, and the results confirmed the histological findings, indicating a reduction in collagen content with combined treatment (Fig 7F). Additionally, assessment of liver function by measuring the level of serum proteins, including ALT, AST, ALB, and ALP, revealed that uridine contributed positively to the recovery of liver function. Notably, its therapeutic effect was comparable to that of PZQ, and co-treatment with uridine and PZQ led to a more pronounced effect (Fig 7I–7J).

## 3. Discussion

The current drug of choice for the treatment of schistosomiasis is PZQ [22]. However, in patients with advanced schistosomiasis, PZQ is no longer effective at reversing the course of liver fibrosis [23]. Therefore, the development of new anti-liver fibrosis drug candidates is highly important. In this study, we explored the effect of uridine on schistosomiasis-induced liver fibrosis and its mechanism by exogenous uridine supplementation in vivo and in vitro. In addition, we demonstrated that the combination of uridine and PZQ in vivo can considerably improve the therapeutic effect of PZQ alone. This study suggests that uridine may be a promising therapeutic intervention for schistosomiasis-induced liver fibrosis and fibrosis-related liver diseases.

The activation of HSCs is a central event in the progression of liver fibrosis [9], and TGF-β is recognized as a profibrotic factor that induces HSC activation [24] and ECM accumulation in a variety of chronic liver diseases [10]. Our previous work demonstrated a notable reduction in liver uridine levels in a mouse model of *S.japonicum*-induced liver fibrosis, which was reflected by decreased uridine phosphorylase 1(UPP1) expression [5]. This finding was further corroborated by TGF-β-induced LX-2 cell activation. Uridine is the most abundant nucleoside in human blood and affects a variety of physiological processes [25]. Recent studies have reported that uridine has anti-inflammatory [26], antifibrosis [27], antioxidation [26] and other effects. In this study, exogenous supplementation with uridine inhibited the proliferation and cell activation of HSCs in vitro, and oral administration of uridine alleviated liver fibrosis in mice with schistosomiasis. Moreover, some studies have shown that the regulation of uridine catabolism can play an antifibrosis role through pharmacological inhibitors of UPP1 [28]. Therefore, regulating the level of uridine or artificially supplementing uridine might be a potential antifibrosis strategy.

Uridine metabolism plays a key role in cellular biochemical processes as it is closely related to other cellular metabolic processes, such as glucose, lipid, and amino acid homeostasis [29]. Previous studies have revealed that there is a crosstalk relationship between uridine metabolism and lipid metabolism [30]. We further studied the relationships among these genes in HSCs. Our findings revealed that activated HSCs had reduced lipid droplets and had lost lipid formation signals, such as SREBP-1c. Exogenous uridine supplementation can promote the activation of lipid formation signals, promote the expression of fatty acid transport and synthetic genes, and increase the formation of intracellular lipid droplets. The overall decline in the fatty acid content of HSCs was consistent with the loss of lipogenic phenotypes, and lipid signaling is important for HSC activation [31]. HSCs undergo transdifferentiation from lipid-storing pericytes to myofibroblastic cells to participate in liver fibrogenesis [31]. Therefore, the ability of uridine to promote the transformation of the HSC lipogenic phenotype may be the key to its anti-liver fibrosis effect.

Notably, emerging evidence suggests that dietary factors significantly modulate uridine levels [32]. For instance, high-fat diets (HFD) may enhance liver regeneration in schistosomiasis models by promoting lipid synthesis pathways [33]. Meanwhile, uridine exacerbates subjective hunger, which in turn drives caloric intake [32]. But in our study, uridine supplementation did not lead to significant changes in body weight in infected mice, indicating that its antifibrotic effects are independent of energy balance disruption. The reason that uridine did not significantly affect the body weight of the mice may be explained by both the fact that the mice were affected by schistosome infection and the inadequate dose of uridine treatment. Future studies should explore whether dietary uridine intake affects schistosomiasis-induced fibrosis progression through similar mechanisms, particularly in high-fat or nutrient-restricted contexts.

Integration of a network database and transcriptomic data was used to predict key targets and pathways of uridine-mediated inhibition of HSC activation. Unexpectedly, the cAMP pathway was found to be enriched. The cAMP pathway is an important signal transduction pathway in cells that is involved in many physiological processes, including metabolic regulation [34], gene expression regulation [34], and cell proliferation and differentiation [35]. Previous studies have shown that the cAMP/PKA/CREB signaling pathway can mediate the activation of HSCs [36]. Our data revealed that the cAMP signaling pathway is activated in activated LX-2 cells and that uridine intervention can inhibit this process. Further use of cAMP inhibitors confirmed that the inhibitory effect of uridine on HSC activation was driven by the cAMP signal. Moreover, during this process, it was observed that the lipogenic phenotype was consistent with the inactivation phenotype of HSCs. The mechanism involed inactivation of the cAMP/PKA/CREB signals, which inhibited the expression of downstream transcription factors such as cFOS and cJUN and initiated the transcription of lipid synthesis signals.

The ADORA1 receptor is part of the G protein-coupled receptor superfamily and is involved in a range of cellular signaling processes [37]. Studies have shown that the ADORA1 receptor can affect intracellular cAMP levels by activating the G protein-coupled signal transduction pathway, thereby regulating related cell functions [38]. In this study, ADORA1 emerged as a potential uridine target based on target prediction and molecular docking analysis. The predicted interaction was experimentally validated by CETSA. While our CETSA results support the physical interaction between uridine and ADORA1, it is important to acknowledge that CETSA assays often require ligand concentrations much higher than physiological levels to produce measurable thermal stabilization effects [39]. Furthermore, ADORA1 knockdown abolished uridine’s ability to suppress fibrotic activation and promote lipid accumulation in LX-2 cells, underscoring the receptor’s essential role in mediating uridine’s antifibrotic effects. These findings are in line with previous studies showing that uridine can reduce arterial pressure through ADORA1 activation [40], and that the hypothermic effects of uridine are mediated by adenosine receptors [41]. Interestingly, uridine increased ADORA1 expression in activated HSCs, suggesting a potential positive feedback mechanism that may enhance receptor-mediated signaling. While this contrasts with the classical desensitization seen in chronic agonist exposure, the long-term effects of sustained uridine treatment remain unclear. Future studies are needed to determine whether prolonged stimulation of ADORA1 may trigger compensatory responses such as receptor internalization or signaling attenuation.

Praziquantel is the drug most extensively used in the treatment of schistosomiasis [42], which encounters marked limitations, primarily because of its inability to remove immature schistosomes and eggs [43]. Consequently, this limitation can result in persistent liver fibrosis in many patients with schistosomiasis because of the remaining eggs [44]. On this basis, we considered combining PZQ with uridine in an attempt to reduce the occurrence of liver fibrosis while treating schistosomiasis. Our study demonstrated that the combined use of PZQ and uridine led to a decrease in the area of advanced liver fibrosis and promoted the recovery of liver function, although it did not lead to a statistically significant reduction in the area of egg granulomas, which suggests that uridine mainly affects fibrosis rather than inflammation.

In addition to its antifibrotic effects, the influence of uridine on schistosome reproductive biology and egg-associated processes remains an open question. Although our current data showed no significant reduction in hepatic egg burden following uridine treatment, it is still possible that uridine may affect egg deposition patterns, antigen composition, or egg migration across host tissues. Previous studies have highlighted the role of host immune responses—particularly Th2-mediated responses—in facilitating egg transit [45], and uridine has been reported to possess immunomodulatory properties [46]. Therefore, future studies will focus on determining whether uridine alters the fecundity of adult worms or modifies the antigenic and migratory behavior of eggs, especially in the context of combination therapy with praziquantel.

**Fig 8:**
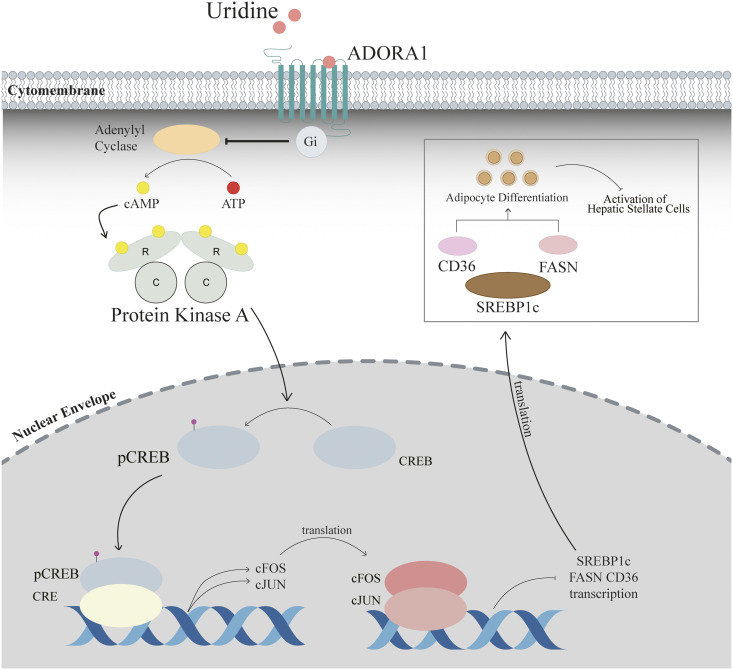
The molecular mechanism of HSC inactivation induced by uridine through regulation of the cAMP/PKA/CREB signaling pathway. The illustration in Fig 8 was drawn by hand using Adobe Illustrator 2021 software.

In conclusion, we elucidated the critical role of uridine in modulating the cAMP/PKA/CREB signaling pathway through ADORA1 receptors, which are involved in the differentiation of lipogenic phenotypes and the activation of HSCs. Additionally, the combination of uridine with PZQ demonstrated increased efficacy in mitigating liver fibrosis induced by schistosomiasis. These findings suggest that uridine has potential as a promising small-molecule therapeutic for the management of schistosomiasis-induced hepatic fibrosis (Fig 8).

## 4. Materials and methods

### 4.1. Ethics approval and consent to participate

The study was approved by the Institutional Review Board (or Ethics Committee) of Jiangsu Institute of Parasitic Diseases (JIPD-2020–002).All animal care and experimental procedures were conducted in strict accordance with the guidelines for the management and use of laboratory animals. No human participants were involved in this research.

### 4.2. Cell Culture and TGF-β Treat

LX-2 cells, which were previously stored in our laboratory, were utilized for this study. The cells were reconstituted from a tube in liquid nitrogen, resuspended in DMEM (HyClone, SH3002201) containing 10% fetal bovine serum (Gibco, 10270106) and 1% penicillin-sulfurstreptomycin, transferred to culture flasks, and incubated at 37°C in an incubator containing 5% CO_2_. The cells were harvested when they reached 80% confluence, and other cell experiments were performed. For TGF-β treatment, LX-2 cells were plated into 6-well plates. After cell attachment, activation of theLX-2 cells was induced by treatment with 25 ng/ml TGF-β (Absin, abs04204) for 12 hours and then the medium was replaced with complete medium containing uridine and/or other reagents.

### 4.3. Evaluation of cellular proliferation

LX-2 cells were plated into a 96-well plate, with triplicate wells assigned for each group. After cell attachment, the medium was replaced with complete medium containing a gradient of uridine concentrations, with 100 μl per well. After 24 hours of treatment, 10 μl of Cell Counting Kit(CCK-8) solution (Vazyme,A311-01) was added to each well, and the plate was incubated in the incubator for 1 hour, followed by absorbance measurement at 450 nm with a microplate reader.

### 4.4. RNA isolation and quantitative PCR

Total RNA was extracted from cells or mouse liver tissues with Freezol (Vazyme, R711) according to the manufacturer’s instructions. The RNA was then reverse transcribed into complementary DNA (cDNA) with a reverse transcription kit (Vazyme, R333). The threshold cycle (Ct) and melting curves were determined with a LightCycler 480 instrument with SYBR Green qPCR Master Mix (Vazyme, Q711-02) and the specified primers (S1 Table). Relative gene expression levels were calculated as fold changes compared with those of the control group by the 2^-ΔΔCt^ method, with β-actin used as the internal reference.

### 4.5. Protein isolation and Western blotting

Cells or tissues were lysed by mixing RIPA lysis buffer (Servicebio, G2002) with a protease inhibitor mixture (Solarbio,P6731) to extract total protein, which was determined with a BCA assay kit (Vazyme, E112-01). SDS‒PAGE was performed with a tris-glycine PAGE gel (4–20%) (TransGen, DG101–01) and tris-gly electrophoresis buffer (Beyotime, P0014B). The proteins were transferred to a PVDF membrane, which was incubated in 5% bovine serum albumin for 60 minutes to block nonspecific binding. The primary antibodies were added to the membrane, which was subsequently incubated at 4°C overnight. The membrane was washed five times with TBST for 10 minutes each before and after incubation with the secondary antibody at room temperature for one hour. High-sensitivity ECL detection was performed with equipment from Cytiva.

### 4.6. Target prediction and reference transcriptome analysis

Assessing uridine targets was carried out through two websites: SwissTargetPrediction (http://www.swisstargetprediction.ch/) and TargetNet (http://targetnet.scbdd.com/). Targets whose probability was greater than 0 were selected as candidates. Cell processing for reference transcriptome analysis was performed as described in Section 4.2. Transcriptome analysis was performed by Oebiotech (Shanghai OE Biotech Co., Ltd.). Subsequent Kyoto Encyclopedia of Genes and Genomes (KEGG) and Gene Ontology(GO) enrichment analyses and other related analyses were completed by the Oebiotech cloud tools platform.

### 4.7. Detection of lipid droplets in LX-2 cells

The accumulation of intracellular lipid droplets was observed with Nile red fluorescent dye (Yuanye,7385-67-3). The storage solution was diluted with PBS at a ratio of 1:1000 to create a Nile red working solution for further use. The methods used for cell inoculation and treatment are described in Section 4.2, except that the uridine treatment experiments were extended to 96 hours. After being washed twice with PBS, the cells were fixed with 4% formaldehyde at room temperature for 30 minutes and flushed three times with double distilled water, and then stained with 1 × Nile red working solution at 37°C for 10 min. After being rinsed with double-distilled water 5 times, the slides were sealed with an anti-fading reagent containing DAPI (Servicebio, G1407). The fluorescence signal was detected under a fluorescence microscope (Nile red Ex/Em = 552/636 nm; DAPI Ex/Em = 364/454 nm).

### 4.8. Immunofluorescence staining detection of PKAc in LX-2 cells

Cell plating and treatment were performed as described in Section 4.2. After the culture medium was aspirated and the cells were washed twice with PBS, fixation was performed with 4% paraformaldehyde at room temperature for 30 minutes, followed by three rinses with double-distilled water. The cells were permeabilized with 0.5% Triton X-100 for 15 minutes and blocked with 5% goat serum for 60 minutes. After being rinsed three times with TBST, the cells were incubated with a rabbit anti-PKAc primary antibody (CST, USA) at 4°C overnight. Before and after incubation with the secondary antibody at room temperature for 1 hour, the cells were rinsed three times with TBST. The slides were mounted with the anti-fade reagent containing DAPI. Observation and imaging were performed under a fluorescence microscope(FITC Ex/Em = 488/525 nm; DAPI Ex/Em = 364/454 nm).

### 4.9. Loading of siRNA into LX-2 cells

The ADORA1-siRNA(si-ADORA1) and control-siRNA(NC-si) (the sequences are shown in S1 Table) were synthesized by Sangon Biotech (China). To load the siRNAs into LX-2 cells, the siRNAs (60 pmol/well) were added to DMEM supplemented with RNA TransMate (Sangon, E607402) and incubated with the cells for 8 hours. Following an incubation period of 48 hours in complete medium, the LX-2 cells were used directly in further experiments.

### 4.10. Cellular Thermal Shift Assay(CETSA)

LX-2 cells were harvested from a 10-cm culture dish, washed twice with PBS, and resuspended in 2 mL of PBS containing a protease inhibitor cocktail (Solarbio, China). The suspension underwent four freeze-thaw cycles (30 seconds in liquid nitrogen, followed by rapid thawing in a 37°C water bath and gentle mixing). After the final thawing, the suspension was sonicated (3 seconds on, 3 seconds off, for 30 cycles). The lysate was clarified by centrifugation at 15000 × g for 20 minutes at 4°C, and the supernatan was collected and stored at -80°C. Protein concentration was measured using a BCA protein assay kit (Biosharp, China).The protein solution was divided into two groups: Control (C group) and Test (T group). The T group was supplemented with uridine (Beyotime, China) to 8 mM, while the C group received PBS. Both groups were incubated at room temperature (25°C) for 2 hours. After incubation, the protein solutions were divided into six tubes and kept on ice. The tubes were heated at six temperatures (40, 50, 60, 70, 80, 90°C) in a preheated metal bath for 5 minutes, then incubated at room temperature for 3 minutes and cooled on ice.After heat treatment, the solutions were centrifuged at 15000 × g for 40 minutes at 4°C to remove denatured proteins. Supernatants were transferred to new tubes for subsequent western blot analysis.

### 4.11. Experimental animals

ICR mice (aged 6 weeks, weighing 20 ± 2 g) were purchased from Zhejiang Viton Lihua Experimental Animal Technology Co., Ltd. (Jiaxing, China) (license number: SCXK (Hangzhou, China) 2019–0001) and kept in the same environment at the Experimental Animal Center of Jiangsu Institute of Parasitic Diseases (license number: SYXK (Su) 2017–0050); the ethical approval number was JIPD-2020–002.

### 4.12. *Schistosoma japonicum* infection

The *Schistosoma japonicum* (Jiangsu strain) used in this study was preserved by the Jiangsu Institute of Parasitic Diseases. The cercariae were obtained from infected Oncomelania snails in our laboratory for use in animal experiments. In both experiments, the abdominal areas of the infection group mice were carefully shaved to ensure proper exposure of the inoculation site. The cercariae were counted under a microscope, and each mouse was contacted with 15 ± 2 cercariae. The infection was performed under controlled conditions (25 °C, 70% humidity) for 15–20 minutes with the slides in contact with the skin. Distilled water was applied to keep the skin moist during the infection process.

### 4.13. Experimental groups and treatment

In the first experiment, 30 mice were divided into two groups: a healthy control group (5 mice) and an infection group (25 mice). The infection group was further subdivided and treated with the following: 50 mg/kg, 100 mg/kg, or 200 mg/kg of uridine, 300 mg/kg praziquantel (PZQ), or no treatment (infected untreated control).

In the second experiment, 30 mice were divided into a healthy control group (10 mice) and an infection group (20 mice). The healthy control group was treated with either 200 mg/kg uridine or no treatment. The infection group received one of the following treatments: 200 mg/kg uridine, 300 mg/kg PZQ, a combination of uridine and PZQ, or no treatment (infected untreated control). Each treatment group consisted of 5 mice.

From the 5th week post-infection, the PZQ treatment group received 150 mg/kg/day of praziquantel (PZQ), dissolved in 1% carboxymethyl cellulose (CMC), by gavage for 3 consecutive days. The control and other treatment groups were administered an equal volume of 1% CMC solution. Starting from the 5th week, mice in the uridine treatment groups received uridine (50, 100, or 200 mg/kg) in drinking water until the end of the study. Water intake was strictly controlled to ensure accurate dosing of uridine, with equal water consumption maintained across all groups. Mice were allowed ad libitum access to water at other times.

### 4.14. Histopathological examination of liver samples

Small pieces of liver tissue from the right lobe of each mouse were fixed in a 4% paraformaldehyde solution for 24 hours. After gradient dehydration, transparency, wax immersion, embedding, sectioning, drying, dewaxing, rehydration, HE staining (Biosharp, BL700B), and Masson staining (Solarbio, G1340), the sections were sealed and observed.

### 4.15. Granulomas number counting and area measurement

The number of granulomas was quantified by counting in five randomly selected low-power fields (magnification ×100) in each liver section. The area of individual egg-induced granulomas was measured using ImageJ software (version 1.54g). All granulomas caused by single eggs in each liver section were analyzed, and the area of each granuloma was calculated by manually delineating the granuloma boundary in ImageJ.

### 4.16. Egg burden assessment

Egg burden was assessed by two approaches. For liver sections, the number of eggs was counted in five randomly selected low-power fields (magnification ×100). For tissue samples, approximately 0.2 g of liver was digested in 10 mL of 5% KOH solution at 37 °C for 12 hours, and a 20 µL aliquot of the digested tissue was transferred to a glass slide for egg counting under a microscope. The egg count was repeated three times for each sample, and the average was calculated.

### 4.17. Detection of hydroxyproline(HYP) content in the liver

Hydroxyproline concentrations were quantified with a hydroxyproline assay kit (Nanjing Jiancheng, A030-2–1). Liver tissue samples were excised and weighed. The tissue was fully hydrolyzed with hydrolysate buffer at 95 °C for 20 minutes. The pH value was adjusted to 6.0–6.8. Activated charcoal was used to clear the supernatant, which was then assayed for HYP content with a colorimetric method with specific reagents. The absorbance was measured at 550 nm, and HYP levels were calculated on the basis of a standard curve.

### 4.18. Mouse serum biochemical assay

Whole blood was collected from the mice, allowed to coagulate at room temperature for 2 hours, and subsequently centrifuged to obtain the serum. Serum levels of aspartate aminotransferase (AST), alanine aminotransferase (ALT), and alkaline phosphatase (ALP) were quantified by the substrate rate double reagent method, whereas albumin (ALB) levels were determined by the endpoint method, with an automatic biochemical analyzer(Beckman Coulter, AU5800).

### 4.19. TR-FRET-based cAMP detection

CHO cells stably expressing human ADORA1 were cultured to ~80% confluence, detached using trypsin, and seeded into 384-well plates at 10 μL/well. A 10-point serial dilution of uridine was prepared in stimulation buffer using an automated liquid handling system (Apricot). Compounds (10 nL/well, 1000 × stock) were dispensed into the assay plate using an ECHO acoustic liquid handler and incubated at 37 °C for 10 minutes. Subsequently, forskolin (final concentration: 200 nM) was added (10 nL/well), and the cells were incubated at 37 °C for an additional 30 minutes. After stimulation, 5 μL/well of Eu-cAMP tracer and 5 μL/well of ULight-anti-cAMP antibody (both diluted to working concentrations in detection buffer) were sequentially added. Plates were centrifuged briefly and incubated at room temperature for 1 hour in the dark. TR-FRET signals were measured using a microplate reader with excitation at 330 nm and emissions collected at 620 nm and 665 nm. The cAMP levels were calculated as the 665/620 nm ratio. EC₅₀ values were derived by nonlinear regression fitting using GraphPad Prism 9.0. All experiments were performed in at least three biological replicates.

### 4.20. Data processing and statistical analysis

The experimental data are presented as the mean± S.D. SPSS 22.0 was used for statistical analysis, and one-way ANOVA and a Dunnett t test were used to compare the significance of differences in data among multiple groups, with a significance level of α = 0.05. A p value <0.05 indicated a statistically significant difference (*p < 0.05, **p < 0.01, ***p < 0.001, ****p < 0.0001). Data visualization was performed with GraphPad Prism 9.0.

## Supporting information

S1 Fig RNA-Seq data analysis. (A) Principal component analysis(PCA) of RNA-Seq data. (B) Statistic of differently expressed gene. (C) Venn of differently expressed gene between control group, TGF-β group and uridine group. (D-F) Volcano map of differently expressed gene between control group, TGF-β group and uridine group. (G-I) GO functional enrichment results of differently expressed gene between TGF-β group and uridine group. (J-K) KEGG enrichment results of differently expressed gene between TGF-β group and uridine group.
(TIF)

S2 Fig Expression ofcAMP signaling pathway in mouse livers. (A) cAMP concentration in mouse livers. (B) Representative Western blot (B) and quantification of CREB protein phosphorylation (C), Phosphokinase A protein c subunit (PKAc) (D), and α-SMA expression in mouse livers. The data represent the mean±S.D from five mice per group. (*P < 0.05, **P < 0.01, ***P < 0.001, ****P < 0.0001).
(TIF)

S3 Fig Changes in body weight and egg burden in mice. (A) Changes in body weight of mice. (B-C) The number of granulomas and eggs in mouse liver sections was observed by H&E staining under a 10x objective lens. (D) Number of eggs per gram of liver. The data represent the mean±S.D from five mice per group. (*P < 0.05, **P < 0.01, ***P < 0.001, ****P < 0.0001).
(TIF)

S4 Fig Morphological analysis of *S. japonicum* eggs in liver tissue from treated and untreated mice. (A-C) Representative light microscopy images of *S. japonicum* eggs in liver sections from (A) infected untreated mice, (B) infected mice treated with uridine (200 mg/kg), and (C) infected mice treated with praziquantel (300 mg/kg). Scale bar = 100 μm.
(TIF)

S5 Fig Lipid storage in the liver of mice. Representative Oil Red O(ORO) staining image of liver sections(A) and quantification of the integrated density(B). Lipid droplets are represented in red and nuclei in blue. The data represent the mean±S.D from five mice per group. (*P < 0.05, **P < 0.01, ***P < 0.001, ****P < 0.0001). The photos in S4A Fig were taken by the author.
(TIF)

S6 Fig Regulation of liver lipid metabolism by uridine in infected mice. (A)MDA levels in mouse livers. (B-F) Relative mRNA expression of cFos, cJun, Srebp1c, Fasn and Cd36 in mouse livers. The data represent the mean±S.D from five mice per group. (*P < 0.05, **P < 0.01, ***P < 0.001, ****P < 0.0001).
(TIF)

S7 Fig Functional validation of uridine as an ADORA1 agonist using cAMP-FRET assay in CHO cells. (A-B) Representative Western blot and quantification of ADORA1 expression in CHO cells. The data represent the mean±S.D. (C) Activation effects of different concentrations of uridine on cAMP levels in wild-type CHO cells. (D) The effect of uridine on the activation of cAMP levels in h-ADORA1 KI CHO cells, showing a dose-response relationship between uridine concentration and the percentage of cAMP activation. The EC50 values are marked with red dashed lines.
(TIF)

S1 Table Sequences of all primers analyzed by real-time PCR. (XLSX)

S2 Table Sequence of the siRNA used in the in vitro experiments. (XLSX)

S3 Table KEGG enrichment analysis of common genes between RNA-Seq and target prediction. (XLSX)
