# Supplementary figures and images for: A novel combination therapy with Uridine and Praziquantel effectively alleviates schistosomiasis-induced hepatic fibrosis through promoting adipogenic differentiation
Source: PLoS Pathog. 2025 Aug 6;21(8):e1013403. doi: 10.1371/journal.ppat.1013403 (PMC12349724; doi:10.1371/journal.ppat.1013403)

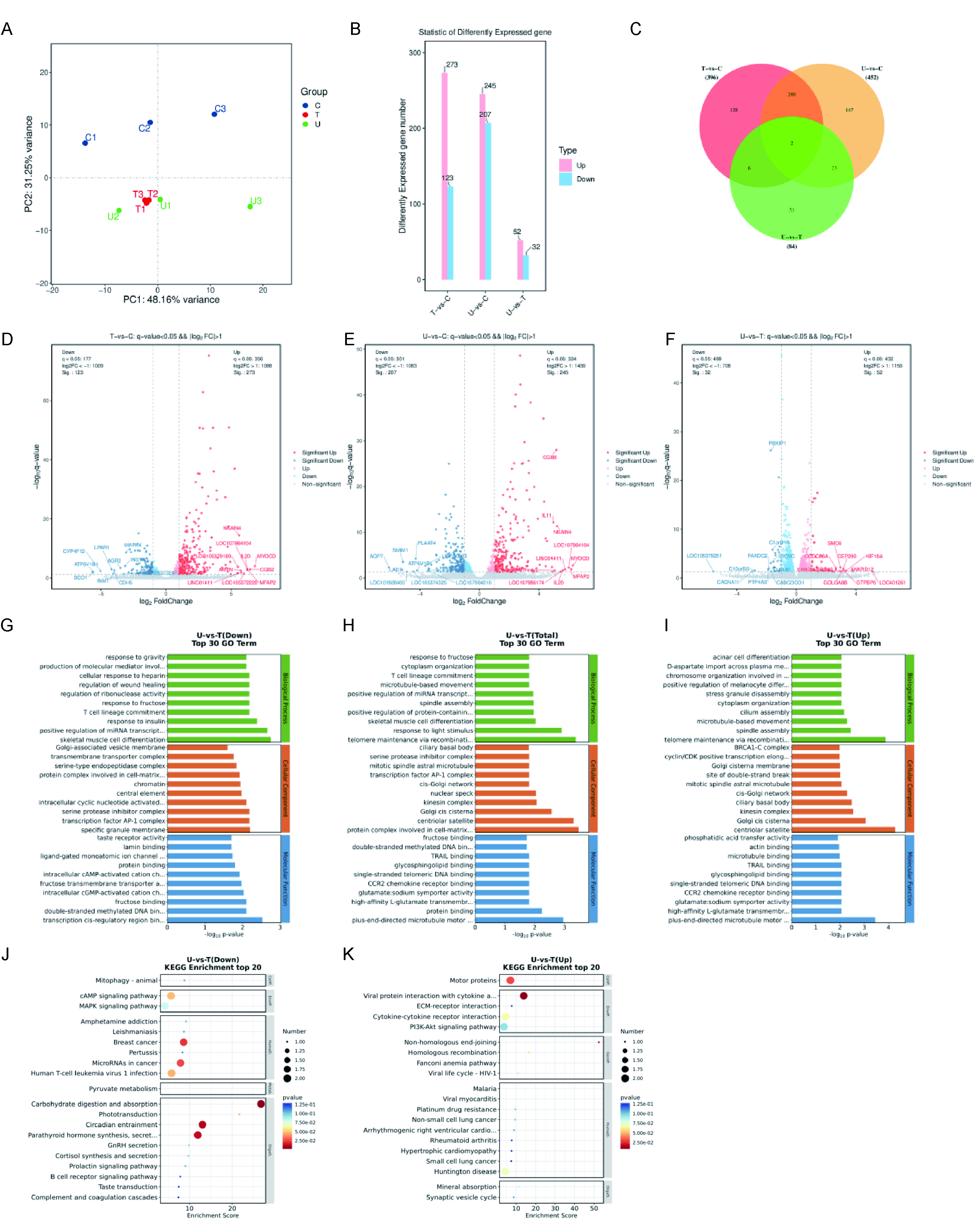

Supplement: S1 Fig — (A) Principal component analysis(PCA) of RNA-Seq data. (B) Statistic of differently expressed gene. (C) Venn of differently expressed gene between control group, TGF-β group and uridine group. (D-F) Volcano map of differently expressed gene between control group, TGF-β group and uridine group. (G-I) GO functional enrichment results of differently expressed gene between TGF-β group and uridine group. (J-K) KEGG enrichment results of differently expressed gene between TGF-β group and uridine group. (TIF) [file ppat.1013403.s001.tif]

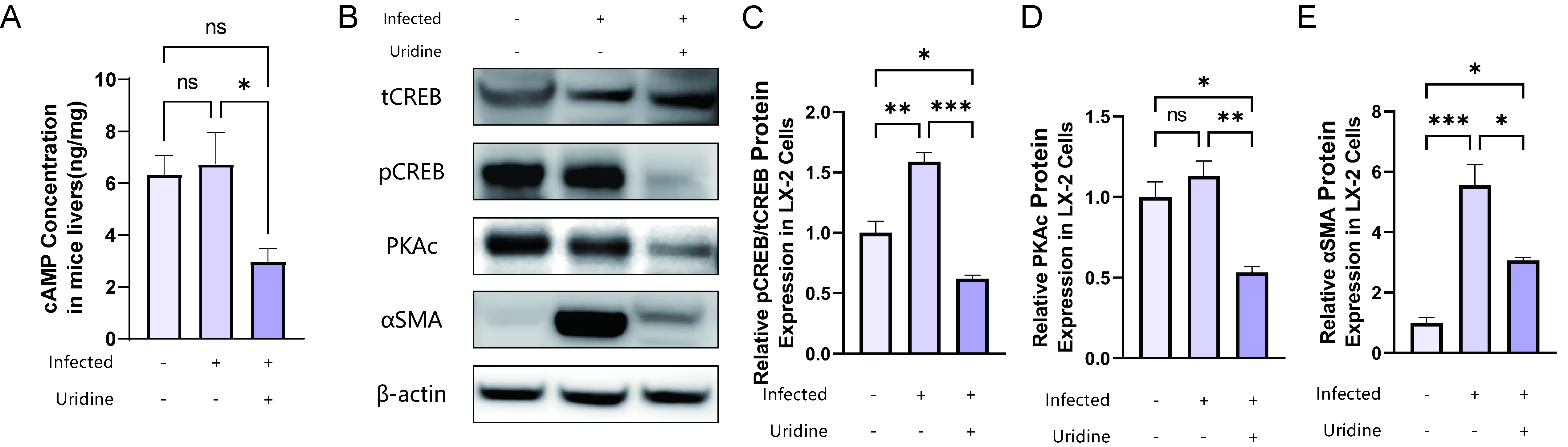

Supplement: S2 Fig — (A) cAMP concentration in mouse livers. (B) Representative Western blot (B) and quantification of CREB protein phosphorylation (C), Phosphokinase A protein c subunit (PKAc) (D), and α-SMA expression in mouse livers. The data represent the mean±S.D from five mice per group. (*P < 0.05, **P < 0.01, ***P < 0.001, ****P < 0.0001). (TIF) [file ppat.1013403.s002.tif]

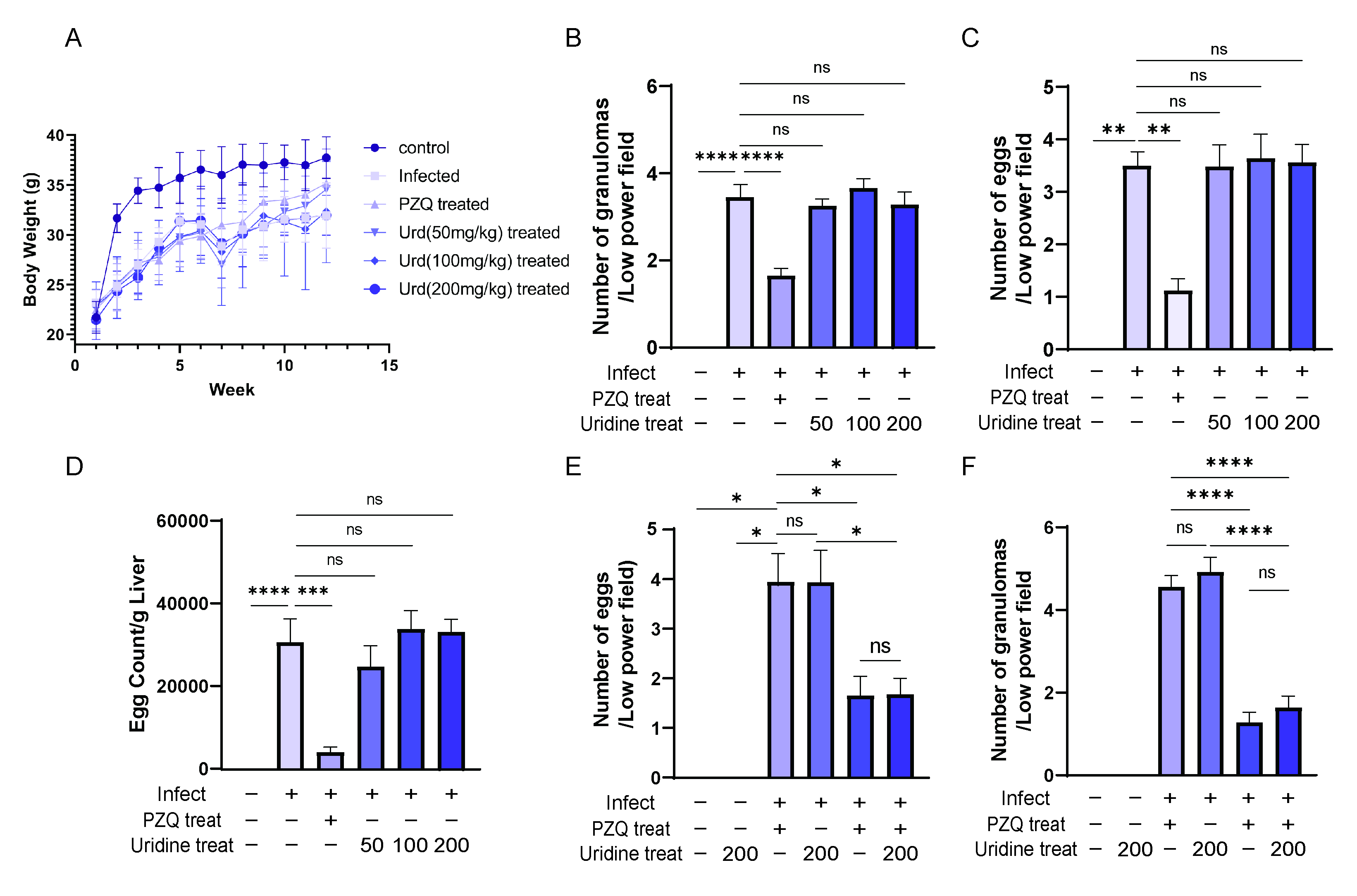

Supplement: S3 Fig — (A) Changes in body weight of mice. (B-C) The number of granulomas and eggs in mouse liver sections was observed by H&E staining under a 10x objective lens. (D) Number of eggs per gram of liver. The data represent the mean±S.D from five mice per group. (*P < 0.05, **P < 0.01, ***P < 0.001, ****P < 0.0001). (TIF) [file ppat.1013403.s003.tif]

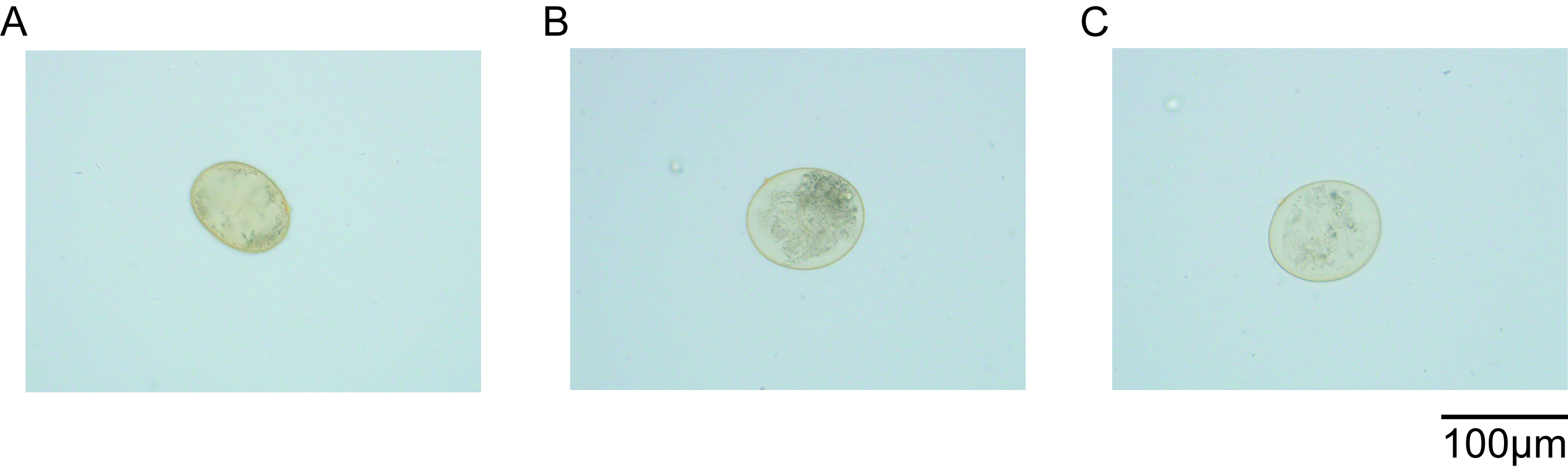

Supplement: S4 Fig — (A-C) Representative light microscopy images of S. japonicum eggs in liver sections from (A) infected untreated mice, (B) infected mice treated with uridine (200 mg/kg), and (C) infected mice treated with praziquantel (300 mg/kg). Scale bar = 100 μm. (TIF) [file ppat.1013403.s004.tif]

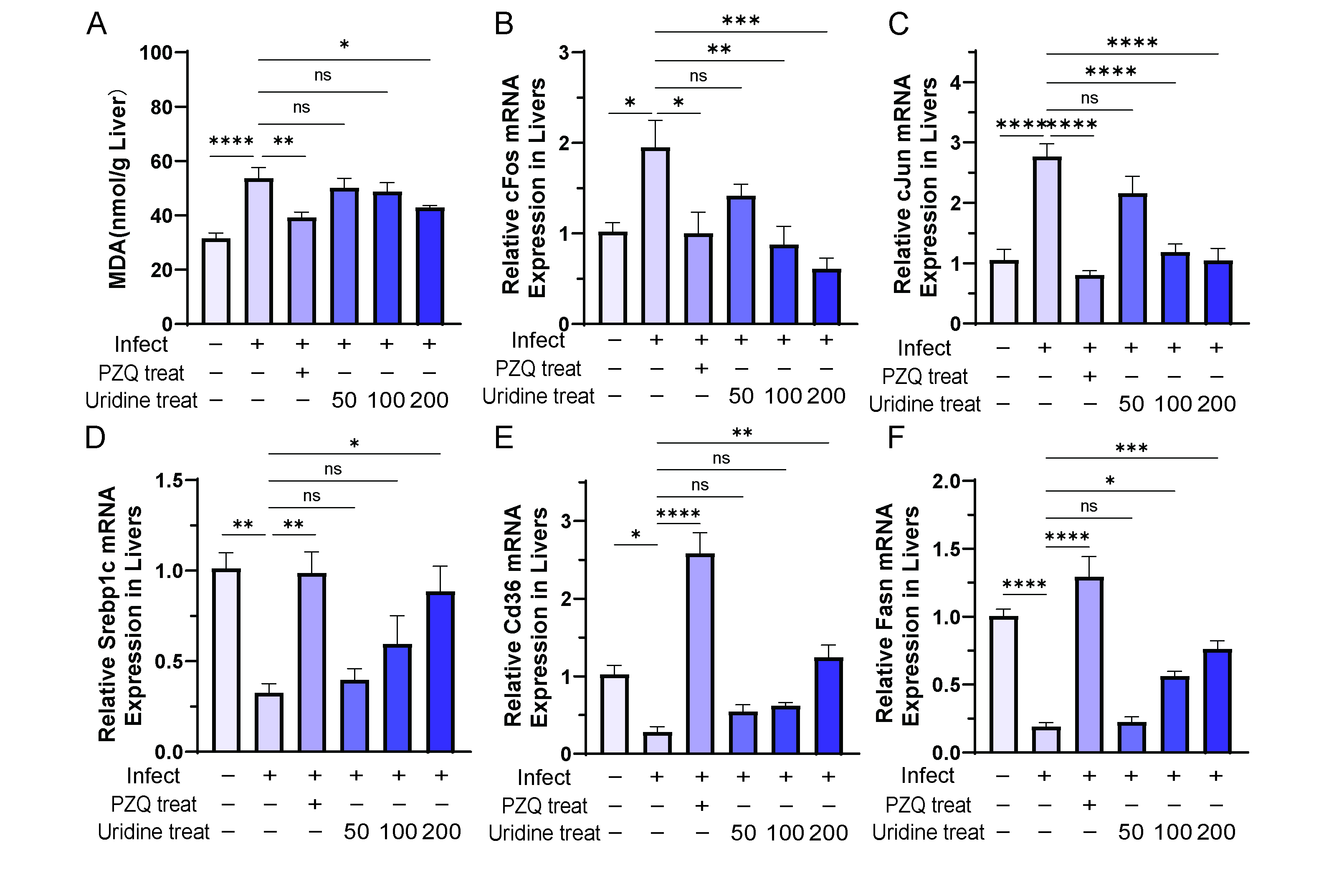

Supplement: S6 Fig — (A)MDA levels in mouse livers. (B-F) Relative mRNA expression of cFos, cJun, Srebp1c, Fasn and Cd36 in mouse livers. The data represent the mean±S.D from five mice per group. (*P < 0.05, **P < 0.01, ***P < 0.001, ****P < 0.0001). (TIF) [file ppat.1013403.s006.tif]

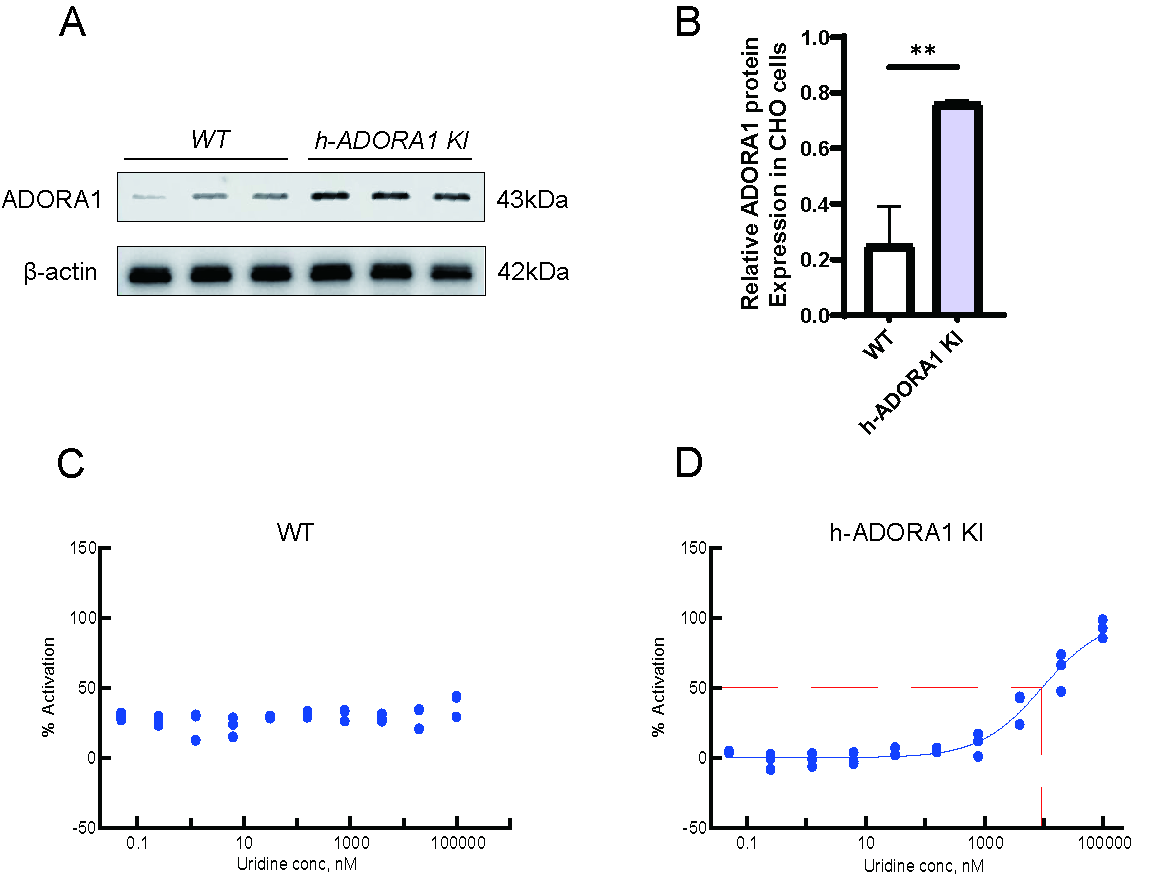

Supplement: S7 Fig — (A-B) Representative Western blot and quantification of ADORA1 expression in CHO cells. The data represent the mean±S.D. (C) Activation effects of different concentrations of uridine on cAMP levels in wild-type CHO cells. (D) The effect of uridine on the activation of cAMP levels in h-ADORA1 KI CHO cells, showing a dose-response relationship between uridine concentration and the percentage of cAMP activation. The EC50 values are marked with red dashed lines. (TIF) [file ppat.1013403.s007.tif]
